# Can acupuncture improve cardiac function in patients after percutaneous coronary intervention? A systematic review and meta-analysis

**DOI:** 10.3389/fmed.2026.1926216

**Published:** 2026-09-09

**Authors:** Zhihong Chen, Jinna Nie, Jiangshan Tian

**Affiliations:** 1 Department of Basic Medicine, Changchun University of Traditional Chinese Medicine, Changchun, Jilin, China; 2 Department of Traditional Chinese Medicine, Shandong University of Traditional Chinese Medicine, Jinan, China

**Keywords:** acupuncture, cardiac function, cardiac remodeling, meta-analysis, percutaneous coronary intervention (PCI)

## Abstract

**Background:**

To systematically evaluate the effects of acupuncture in improving cardiac function and the reported adverse events in patients after percutaneous coronary intervention (PCI) through meta-analysis.

**Methods:**

Electronic databases including PubMed, China National Knowledge Infrastructure, Wanfang Medical, China Biomedical Literature Database, VIP Database, Embase, Ovid Medline, Sinomed Database and the Cochrane Library were searched from inception to May 2026. Two reviewers independently screened the literature according to the inclusion and exclusion criteria and extracted data. The methodological quality was assessed using the Cochrane Handbook for Systematic Reviews of Interventions (version 5.1), and meta-analysis was performed with Review Manager 5.3. The evidence was appraised per the GRADE framework.

**Results:**

A total of 8 RCTs involving 759 patients were included, with 379 patients in the acupuncture group (247 males and 132 females) and 380 in the control group (conventional treatment or sham acupuncture; 257 males and 123 females). The results of the meta-analysis showed that, revealing that acupuncture was associated with better outcomes than conventional treatment or sham acupuncture in several measures. Specifically, the intervention led to a substantial boost in left ventricular ejection fraction (LVEF) by an average of 4.26 percentage points [95% CI (1.21, 7.31), *P* = 0.006], while also enhancing functional capacity as evidenced by a 43.88-m improvement in the 6-minute walk distance [95% CI (10.40, 77.36), *P* = 0.01]. On the biochemical front, acupuncture demonstrated its efficacy by reducing serum creatine kinase-MB (CK-MB) levels [SMD = −1.99, 95% CI (−3.46, −0.52), *P* = 0.008]. The treatment also showed favorable effects on cardiac remodeling, evidenced by decreased left ventricular end-systolic diameter (LVESD) by 2.56 mm [95% CI (−4.65, −0.46), *P* = 0.02] and left ventricular end-diastolic diameter (LVEDD) by 1.36 mm [95% CI (−2.34, −0.37), *P* = 0.007]. Additionally, acupuncture was associated with a lower risk of major adverse cardiovascular events (MACE) by 60% [RR = 0.40, 95% CI (0.24, 0.66), *P* = 0.0005], positioning it as a promising adjunctive therapy for cardiovascular management.

**Conclusion:**

Current evidence suggests that acupuncture may improve cardiac function in patients after PCI. However, the available literature is limited in both quantity and methodological quality. More rigorously designed, large-sample, high-quality RCTs are warranted to further validate these findings and provide more robust evidence for evidence-based practice.

**Systematic Review Registration:**

identifier CRD420261410954.

## 1 Introduction

Cardiovascular disease remains a leading cause of morbidity and mortality among the elderly population (1). Coronary artery disease (CAD) is the leading cause of cardiovascular morbidity and mortality (2). The fundamental pathology is coronary atherosclerosis. During an attack, patients may present with chest tightness, chest pain, and arrhythmias; in severe cases, heart failure or even sudden death can ensue. Currently, the global prevalence and mortality rates of cardiovascular disease are still on a continuous rise (3). Percutaneous coronary intervention (PCI) is a nonsurgical, minimally invasive interventional procedure that aims to relieve coronary stenosis or occlusion and restore blood supply to the ischemic myocardium (4). Clinically, PCI is a vital and widely adopted treatment for coronary heart disease. In particular, for all patients with acute ST-elevation myocardial infarction (STEMI) presenting within 12 h of symptom onset, primary PCI is the preferred reperfusion strategy, as it can rapidly recanalize the infarct-related artery and significantly reduce acute-phase mortality (5). PCI is also indicated for non-ST-elevation acute coronary syndrome (NSTE-ACS), unstable angina, and stable angina. Although the overall prognosis after PCI is favorable, the entire procedure—from vascular access to the intervention itself—carries a risk of injury to the access vessel and coronary arteries inflicted by related devices. This can lead to severe complications, including coronary embolism, no-reflow phenomenon, periprocedural major bleeding, periprocedural myocardial injury, and myocardial infarction (6), Among geriatric patients, this mechanistic risk is compounded by age-driven physiological attrition, frailty, multimorbidity, polypharmacy, and diminished intrinsic capacity (7). These converging factors jointly predispose elderly individuals to excess postoperative complications and early readmission. Therefore, post-PCI rehabilitation management is of paramount importance.

Acupuncture (including electroacupuncture, transcutaneous electrical acupoint stimulation, etc.), as an innovative and cost-effective modality for preventing and treating cardiovascular diseases, has been increasingly adopted by healthcare professionals (8). Studies have demonstrated that acupuncture can alleviate myocardial ischemia and myocardial ischemia-reperfusion injury (MIRI), thereby improving cardiac function (9–11). The underlying mechanisms are associated with attenuating the inflammatory response, combating oxidative stress, regulating cardiomyocyte autophagy, and inhibiting apoptosis (12–14).

To date, although several primary studies have explored the beneficial effects of acupuncture on cardiac function in post-PCI patients, a systematic review and meta-analysis that synthesizes the relevant clinical evidence is still lacking. Therefore, this meta-analysis was conducted to systematically assess the effects on cardiac function and the reported adverse events of acupuncture in patients after PCI based on the currently available evidence. The ultimate goal is to reduce the incidence of post-PCI complications and mortality, alleviate patient suffering and anxiety, reduce hospital duration, alleviate financial strain on medical facilities and provide a reference for modern clinical decision-making.

## 2 Methods

This research paper was put together and presented following the gold standard guidelines laid out in the PRISMA statement. What's more, we got the ball rolling by registering our game plan ahead of time in PROSPERO, the international database for systematic review protocols. (registration number: CRD420261410954).

### 2.1 Search strategy

A systematic literature search was conducted in China National Knowledge Infrastructure, VIP Database, China Biomedical Literature Database, Wanfang Medical, Embase, PubMed, Ovid, and the Cochrane Library for studies available from database inception to May 2026.The search terms included (“acupuncture” OR “electroacupuncture” OR “acupoint” OR “warm needling” OR “acupoint injection” OR “fire acupuncture” OR “wrist-ankle acupuncture” OR “auricular acupuncture”) AND (“Percutaneous Coronary Intervention” OR “Percutaneous Coronary Revascularization” OR “Coronary Interventions, Percutaneous” OR “Interventions, Percutaneous Coronary” OR “PCI” OR “after coronary stenting” OR “after coronary artery intervention surgery” OR “coronary heart disease”). Details of the search strategy are provided in the Supplementary Table S1.

### 2.2 Eligibility criteria

Inclusion criteria:

(a) Study type: randomized controlled trials (RCTs) evaluating acupuncture therapy in patients after PCI, with no language limitation.
(b) Participants: patients with acute coronary syndrome (ACS) who had undergone successful PCI, including those with post-PCI cardiac dysfunction. Diagnostic criteria were based on relevant clinical guidelines (6, 15, 16). No restrictions were imposed regarding sex, age, or nationality.
(c) Interventions: the experimental group received acupuncture therapy (including manual acupuncture, electroacupuncture, warm needling, fire acupuncture, auricular acupuncture, acupoint injection, and other invasive acupuncture modalities) in addition to conventional treatment. The control group received conventional treatment alone or conventional treatment plus sham acupuncture (needling at non-acupoints).
(d) Eligible studies were required to report at least one of the following outcomes: left ventricular ejection fraction (LVEF), left ventricular end-systolic diameter (LVESD), left ventricular end-diastolic diameter (LVEDD), serum creatine kinase-MB (CK-MB), 6-minute walk distance (6MWD), or the incidence of major adverse cardiovascular events (MACE).

Exclusion criteria include:

(a) Studies with complex interventions, such as acupuncture combined with other Chinese medicine therapies (e.g., oral Chinese herbal formulas, Chinese patent medicines).
(b) Studies for which the full text was unavailable.
(c) Non-RCTs, animal experiments, systematic reviews, narrative reviews, mechanistic studies, and conference abstracts.
(d) Studies with incomplete outcome data.

### 2.3 Data screening and extraction

We used the Zotero reference management tool to organize retrieved records, weeding out any duplicate entries in the process. Two reviewers conducted independent literature screenings: first, they assessed titles and abstracts to narrow down the pool, then moved on to full-text evaluations. After that, the same pair of reviewers used a pre-designed, standardized data extraction form to pull information from the selected studies, working independently the whole time. The data collected included details like lead author, publication year, sample size, intervention types, and outcome metrics. If the reviewers hit a snag or disagreed at any stage—whether during screening or data extraction—they brought in the corresponding author to settle the dispute and reach a consensus.

### 2.4 Literature quality evaluation and certainty assessment

Assessment of study inclusion standards adhered to the Cochrane Handbook for Systematic Reviews, 5.1, and the Risk of Bias Assessment Tool, 2.0. The assessment included five domains: bias related to the randomization process, deviations from intended interventions, missing outcome data, outcome measurement, and selection of the reported results.

The GRADE methodology was applied to ascertain the reliability of evidence, taking into account indirectness, imprecision, risk of bias, inconsistency, publication bias, and other considerations. The overall evidence was then rated as “high”, “moderate”, “low”, or “very low”.

### 2.5 Statistical analysis

Statistical analyses were performed using Review Manager (RevMan) version 5.3 software. For continuous variables, mean differences (MD) or standardized mean differences (SMD) with 95% confidence intervals (CI) were used to estimate pooled effect sizes. For dichotomous variables, risk ratios (RR) or odds ratios (OR) with 95% CIs were calculated to assess pooled effects. Heterogeneity among the included studies was examined using the Chi-square test (*α* = 0.10) together with the *I*^2^ statistic. When clinical and methodological homogeneity was sufficient and statistical heterogeneity was low (*P* ≥ 0.10 and *I*^2^ ≤ 50%), a fixed-effect model was employed for meta-analysis. If substantial heterogeneity was detected (*P* < 0.10 or *I*^2^ > 50%), potential sources were explored through subgroup analyses or sensitivity analyses, and a random-effects model was applied for aggregating the effect size estimations. If a single study exerted an excessively large weight in the pooled analysis, sensitivity analyses were conducted to assess the robustness of the results. When heterogeneity was too high to allow meaningful quantitative synthesis, a descriptive analysis was performed instead. In view of the fact that no outcome comprised ten or more studies, and considering the recognized unreliability and insufficient power of formal publication bias tests (e.g., funnel plots or Egger's regression) with small study numbers, which can lead to misleading inferences, we elected not to conduct such evaluations.

## 3 Results

### 3.1 Literature search

**Figure 1:**
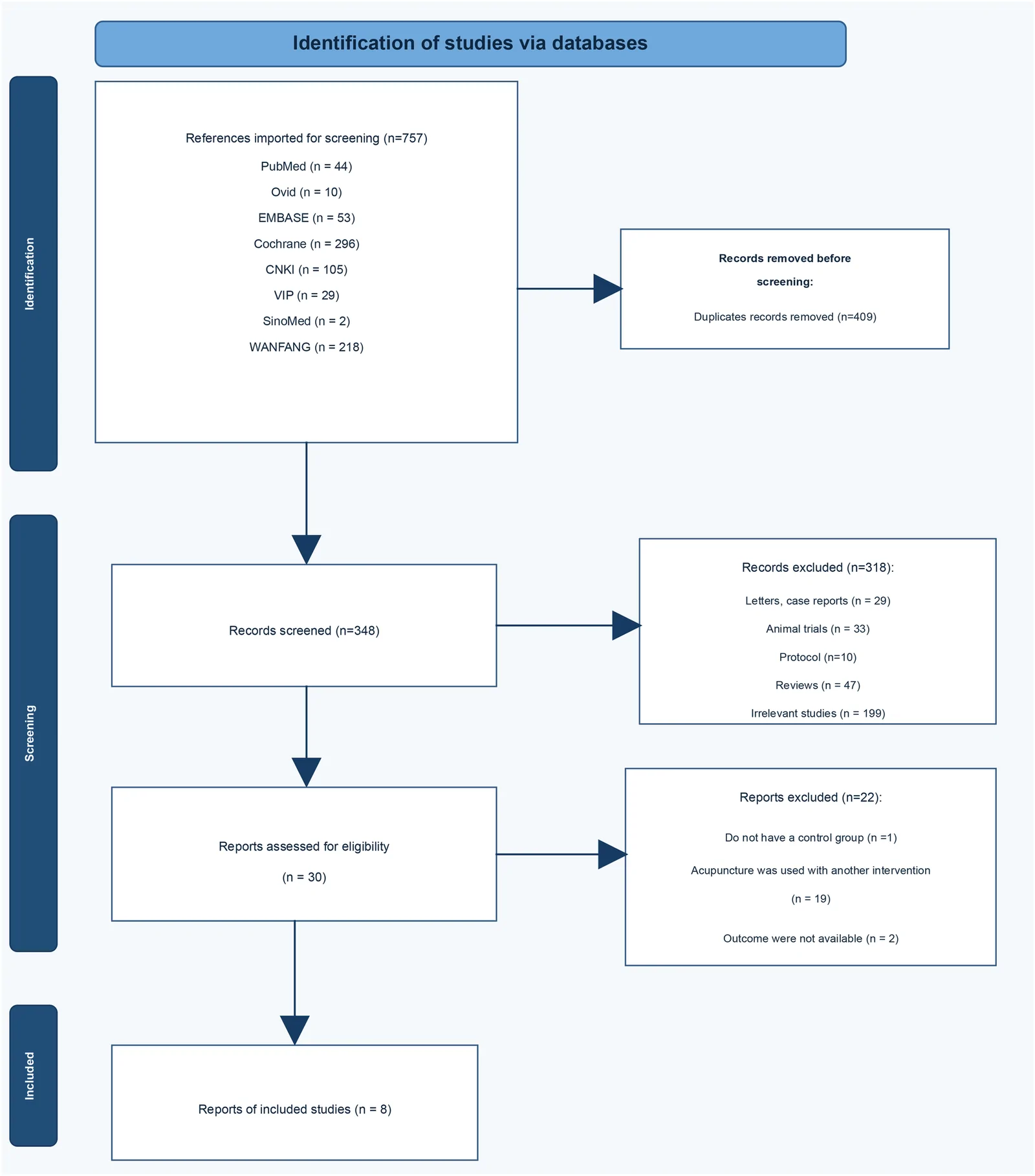
PRISMA flow diagram.

Our search strategy initially surfaced 757 records, which we whittled down to 348 after eliminating duplicates. Two independent reviewers then dove into the titles and abstracts, casting aside 318 articles that didn't quite hit the mark for our review—a process where they saw eye to eye, evidenced by a kappa score of 0.832. This left us with 30 papers that looked promising enough for a deep dive. After poring over the full texts, we had to give the thumbs down to 22 more articles, though this time the reviewers were completely in sync, hitting a perfect kappa score of 1.000. In the end, we were left with 8 studies that made the cut, encompassing 759 patients in total—379 in the acupuncture group (247 guys and 132 gals) and 380 in the control group (who received either conventional treatment or sham acupuncture; 257 guys and 123 gals). The sample size of individual studies ranged from 21 to 102 patients. Dropouts were reported in four studies, while the remaining studies did not report any loss to follow-up or withdrawals (17–20). The excluded studies and the corresponding reasons for exclusion are presented in Supplementary Table S3. Figure 1 maps the literature screening workflow, while Tables 1, 2 outline the core demographic and methodological traits of the eligible research studies.

**Table 1:** Study characteristics.

Author and Year	Age mean (SD)		Sample size (male/female)		Intervening measure		Outcomes
	T	C	T	C	T	C	
Du 2023 (17)	62.31 ± 9.92	60.77 ± 10.78	39 (30/9)	40 (28/12)	ACU + CT	CT	②③⑤
Li 2020 (21)	57.36 ± 6.03	56.82 ± 6.37	48 (27/21)	48 (25/23)	EA + CT (+intravenous injection)	CT (+intravenous injection)	①②③④⑥
Liang 2021 (22)	64.16 ± 5.28	63.79 ± 5.36	43 (24/19)	43 (23/20)	ACU + CT	CT	①②③
Shao 2024 (18)	56.7 ± 11.2	54.6 ± 10.6	21 (16/5)	21 (14/7)	ACU + CT	CT	①⑥
Wang 2015 (19)	60.69 ± 9.70	59.07 ± 9.46	102 (67/35)	102 (81/21)	Intraoperative EA	Sham stimulation	①②⑥
Wu 2026 (20)	56.55 ± 7.83	56.95 ± 7.87	38 (29/9)	38 (27/11)	ACU + CT	CT	①②③⑤⑥
Yang 2023 (23)	62.21 ± 8.82	62.52 ± 8.76	50 (27/23)	50 (28/22)	ACU + CT	CT	①②④
Zeng 2021 (24)	59.6 ± 11.5	59.0 ± 8.3	38 (27/11)	38 (31/7)	ACU + CT	CT	⑤

C, control group; T, experimental group; ACU, acupuncture; CT, conventional treatment; EA, electroacupuncture; ①, LVEF; ②, LVEDD; ③, LVESD; ④, CK-MB; ⑤, 6MWD; ⑥, MACE.

**Table 2:** List of specific acupuncture intervention measures included in the study.

Author and Year	Acupoint selection method	Acupuncture Method and Parameters	Time of therapy
Du 2023 (17)	BL15, CV14, SP4, PC6 (laterality not specified)	MA with filiform needle; 30 min/QOD	4 weeks
Li 2020 (21)	Bilateral PC6	EA using Han's Acupoint Nerve Stimulator (HANS) at 100 Hz; intensity adjusted to patient tolerance; 30 min/QD × 5d	5 days
Liang 2021 (22)	Bilateral PC6, SP4, CV17, SP10, ST36, SP6	Perpendicular insertion with 0.30 mm × 40 mm filiform needles to a depth of 0.5–1 cun; after deqi, needle manipulation for 1 min; 30 min/QD	2 weeks
Shao 2024 (18)	Huatuo Jiaji points (bilateral from T1 to L5, 0.5 cun lateral to posterior midline; 34 points in total)	Perpendicular insertion with filiform needles to a depth of 0.5–0.8 cun; after deqi, needles retained for 30 min with manipulation every 10 min; treatment suspended during menstruation; 3/week	8 weeks
Wang 2015 (19)	Bilateral PC6, PC4	EA applied 1–2 h before PCI; 30min	1 day before PCI (once preoperatively)
Wu 2026 (20)	PC6, HT7, CV6, SP10, ST36 (laterality not specified); CV17	Insertion with 0.25 mm × 25 mm or 40 mm filiform needles; after deqi, PC6 and HT7 connected to KWD-808I EA device (Yingdi) with 2/100 Hz dense-disperse wave; intensity to patient tolerance; 30 min;3–4/week	8 weeks
Yang 2023 (23)	PC6, HT7, Xiongtong point (chest pain point), HT6 (laterality not specified)	MA with filiform needles to achieve deqi; 30 min;BID; 6/week	2 weeks
Zeng 2021 (24)	PC6, PC7, LU9; additional points: SP10, LR3, GV9 (laterality not specified)	Insertion with 0.25 mm silver needles; after deqi, connected to EA device at 15–20 Hz, 2–4 mA; 30 min;once daily for 2 days preoperatively and once daily for 14 days postoperatively	2 days before PCI and 14 days after PCI

Acupoints in this table are presented using the standard international codes recommended by the World Health Organization (WHO) for acupuncture point nomenclature. The corresponding Chinese point names are as follows Xinshu (BL15); Juque (CV14); Gongsun (SP4); Neiguan (PC6); Danzhong (CV17); Xuehai (SP10); Zusanli (ST36); Sanyinjiao (SP6); Ximen (PC4); Shenmen (HT7); Qihai (CV6); Xuehai (SP10); Yinxi (HT6); Daling (PC7); Taiyuan (LU9); Taichong (LR3); Zhiyang (GV9); MA, manual acupuncture; EA, electroacupuncture; QD, quaque die; QOD, quaque altera die; BID, bis in die.

### 3.2 Quality assessment and certainty assessment

The risk of bias of the eight included RCTs was reassessed using the revised Cochrane risk-of-bias tool for randomized trials (RoB2.0), covering five domains: bias arising from the randomization process, bias due to deviations from intended interventions, bias due to missing outcome data, bias in measurement of the outcome, and bias in selection of the reported result.

For the randomization process, one study (19) was judged to have a low risk of bias because it reported both random sequence generation and adequate allocation concealment. Six studies (17, 18, 20–22, 24) were rated as having some concerns, mainly because random allocation was reported but allocation concealment was insufficiently described. One study (23) was judged to have a high risk of bias because it did not clearly report randomization and appeared to assign patients according to the treatment regimen. For deviations from intended interventions, all studies were judged to be at low risk, as no important deviations from the assigned interventions or imbalance in co-interventions were reported. In cases of absent outcome data, the studies were deemed to pose a low risk due to the availability of comprehensive primary outcome information. with no relevant missing participant data or with dropouts fully documented and balanced between groups.

**Figure 2:**
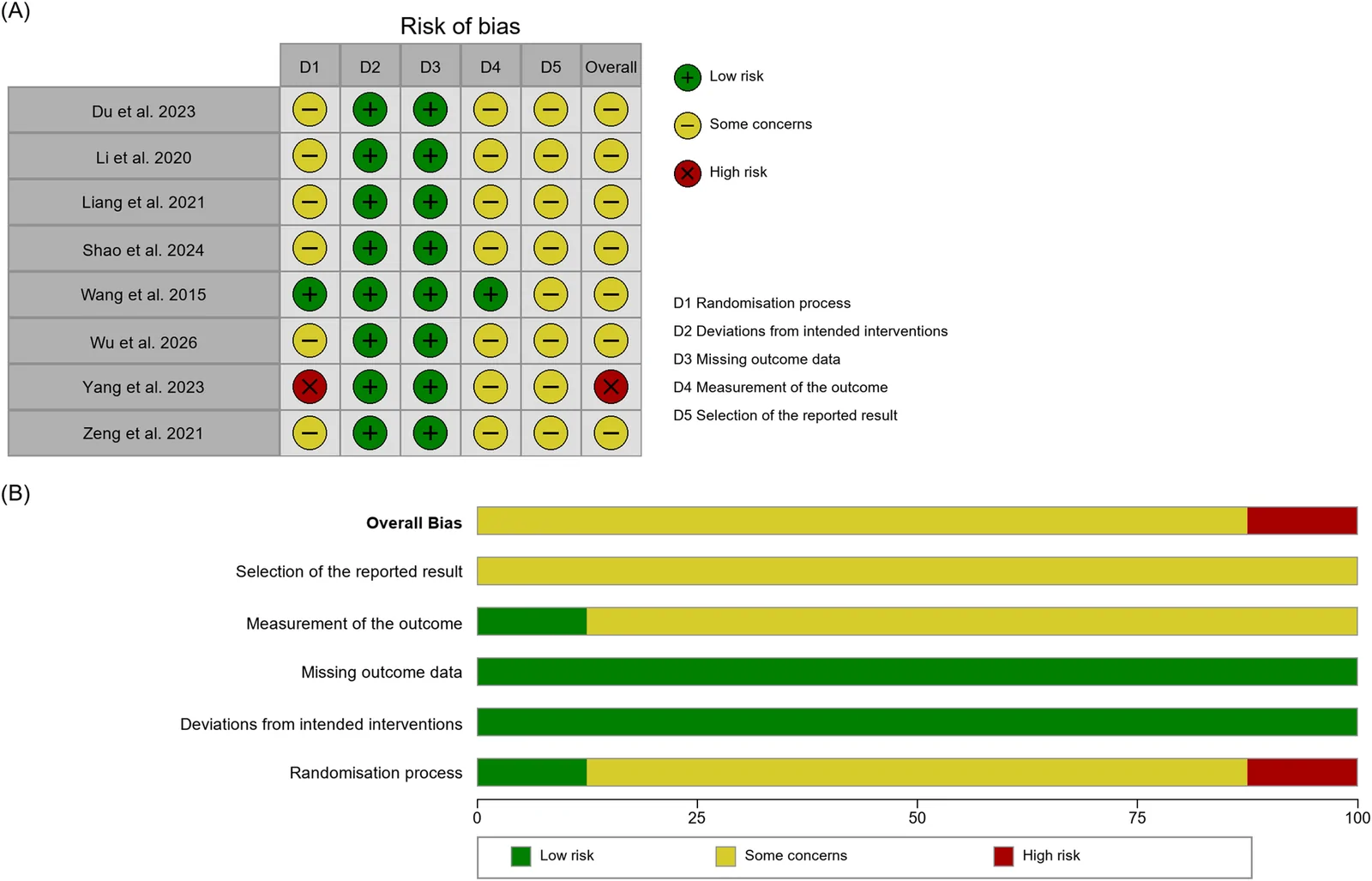
Risk of bias assessment using the Cochrane RoB 2.0 tool. **(A)** Risk of bias. **(B)** Overall Bias.

For outcome assessment procedures, one study (19) was rated as low risk because outcome assessor blinding was clearly reported, wherein the other research presented certain reservations owing to inadequate details regarding assessor masking (given that blinding of the acupuncturist is inherently impossible in acupuncture trials, only single-blinding was feasible). For selection of the reported result, all studies were judged to have some concerns because prespecified protocols or statistical analysis plans were generally unavailable, making it difficult to determine whether the reported outcomes were selected from multiple measurements or analyses. Collectively, seven studies had moderate concerns and one demonstrated significant bias potential. The detailed RoB 2 assessment is presented in Figure 2.

### 3.3 Results of primary outcomes

#### 3.3.1 LVEF

**Figure 3:**
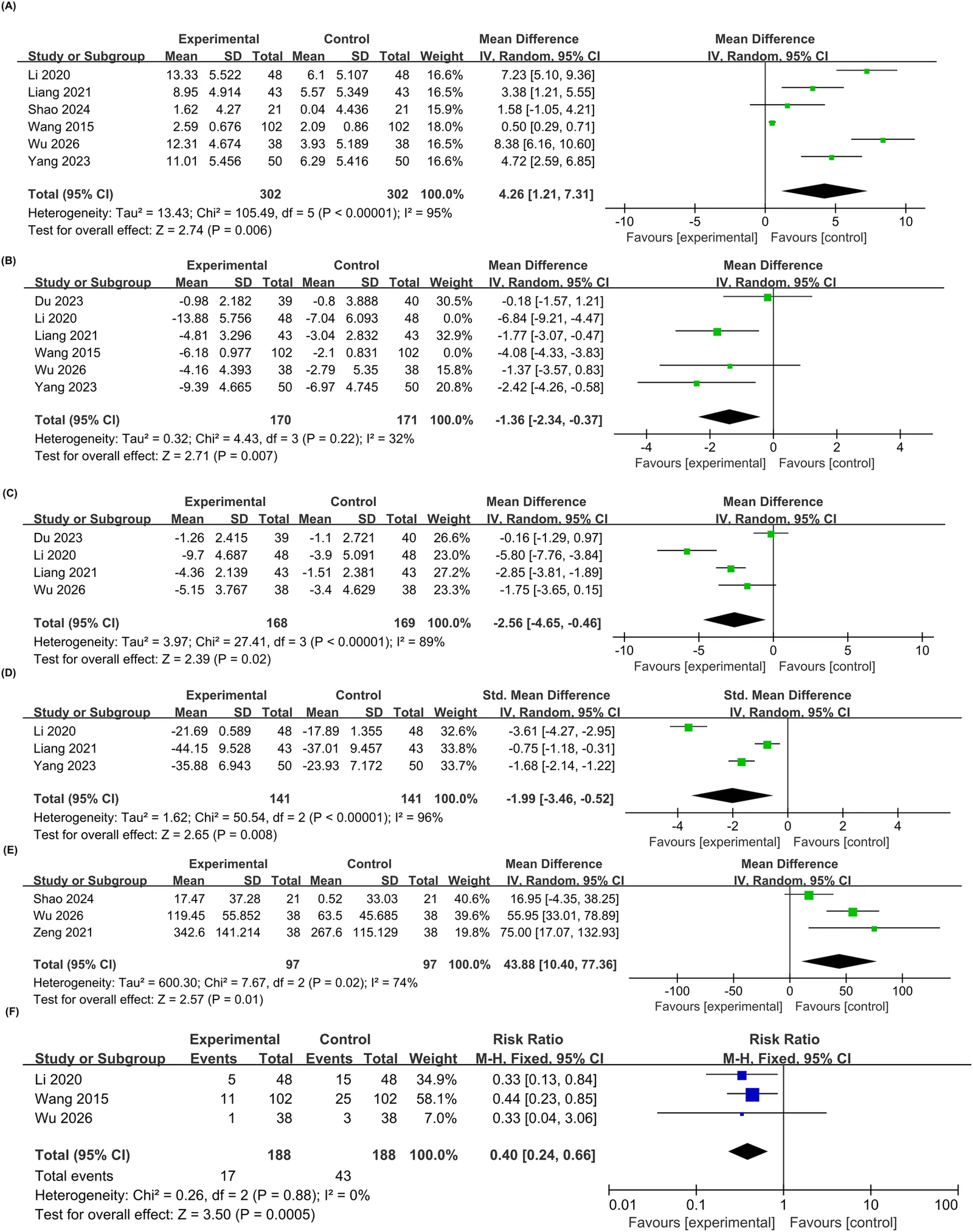
The baseline and post-treatment forest plots. **(A)** The effect of LVEF. **(B)** The effect of LVEDD. **(C)** The effect of LVESD. **(D)** Serum CK-MB levels. **(E)** The effect on increasing 6MWD. **(F)** The incidence of MACE.

Six studies involving 604 patients (302 in the acupuncture group and 302 in the control group) reported the effect of acupuncture vs. control on LVEF. Heterogeneity assessment revealed substantial statistical heterogeneity (*P* < 0.00001, *I*^2^ = 95%). Sensitivity analyses performed by omitting each study individually failed to eliminate the heterogeneity, prompting subgroup analyses stratified by acupuncture modality. The manual acupuncture subgroup (3 studies) showed low heterogeneity (*P* = 0.19, *I*^2^ = 39%), whereas the electroacupuncture subgroup (3 studies) remained highly heterogeneous (*P* < 0.00001, *I*^2^ = 98%). Therefore, a random-effects model was used. The collective findings revealed that patients receiving acupuncture demonstrated a superior enhancement in left ventricular ejection function compared to the control group, with a mean difference of 4.26 [95% CI (1.21, 7.31), *P* = 0.006], as illustrated in Figure 3A.

#### 3.3.2 LVEDD

Six studies involving 641 patients (320 in the acupuncture group and 321 in the control group) reported the effect on LVEDD reduction. The heterogeneity assessment indicated substantial statistical heterogeneity (*P* < 0.00001, *I*^2^ = 91%). Sensitivity analysis was performed by sequentially omitting each study, and after excluding two studies (Wang and Li), the heterogeneity decreased to *P* = 0.22, *I*^2^ = 32%. Consequently, a fixed-effect model was utilized for the meta-analysis. The aggregated findings revealed that the acupuncture group experienced a larger decrease in LVEDD compared to the control group [MD = −1.36, 95% CI (−2.34, −0.37), *P* = 0.007], as illustrated in Figure 3B.

#### 3.3.3 LVESD

Four studies with 337 patients (168 in the acupuncture group and 169 in the control group) reported the efficacy on reducing LVESD. A high degree of statistical heterogeneity was observed (*P* < 0.00001, *I*^2^ = 89%). Sensitivity analyses, performed by iteratively omitting individual studies, failed to resolve the substantial heterogeneity; Consequently, a random-effects model was utilized. The consolidated data indicated that the acupuncture cohort exhibited a notably higher decrease in LVESD than the control cohort [MD = −2.56, 95% CI (−4.65, −0.46), *P* = 0.02], as depicted in Figure 3C.

#### 3.3.4 CK-MB

Three studies involving 282 patients (141 in the acupuncture group and 141 in the control group) reported the effect on reducing serum CK-MB levels. Significant statistical heterogeneity was detected (*P* < 0.00001, *I*^2^ = 96%); Among them Liang and Yang measured CK-MB using enzymatic activity units (U/L), whereas Li used mass concentration units (ng/mL). Although the use of SMD may partly reduce the impact of unit differences, variations in measurement methods and variance structures may still have contributed to the substantial heterogeneity. Given that only three studies were available for this outcome, the reliability and interpretability of quantitative pooling were limited. Therefore, we synthesized the findings narratively rather than conducting a formal meta-analysis. Overall, the included studies suggested that acupuncture was associated with a greater reduction in CK-MB levels than the control intervention as shown in Figure 3D.

#### 3.3.5 6MWD

Three studies involving 194 patients (97 in the acupuncture group and 97 in the control group) reported the effect on increasing 6-minute walk distance (6MWD). Notably, considerable statistical heterogeneity emerged across the studies (*P* = 0.02, *I*^2^ = 74%), prompting the adoption of a random-effects model for analysis. Zeng et al. assessed 6MWD at 14 days post-surgery, while Wu and Shao measured it after eight weeks of treatment. This discrepancy in assessment time points likely explains the elevated heterogeneity. The combined findings revealed that patients receiving acupuncture treatment made substantially greater strides in their 6-minute walking distance compared to the control group [MD = 43.88, 95% CI (10.40, 77.36), *P* = 0.01], as illustrated in Figure 3E.

#### 3.3.6 MACE

Three studies involving 376 patients (188 in the acupuncture group and 188 in the control group) reported the incidence of major adverse cardiovascular events (MACE) following PCI, including serious arrhythmia, recurrent angina, bleeding, bradycardia, and hypotension. In total, 17 MACE occurred in the acupuncture group compared with 43 in the control group. No statistical heterogeneity was detected (*P* = 0.88, *I*^2^ = 0%); therefore, a fixed-effect model was applied. The consolidated data revealed a notably decreased occurrence of MACE within the acupuncture cohort compared to the control group [RR = 0.40, 95% CI (0.24, 0.66), *P* = 0.0005], as depicted in Figure 3F.

### 3.4 Sensitivity analysis

To evaluate the robustness of the pooled results and further explore potential sources of heterogeneity, sensitivity analyses were conducted for outcomes with substantial heterogeneity or those susceptible to the influence of individual studies. Taking LVEDD as an example, the original pooled estimate indicated that acupuncture reduced left ventricular end-diastolic diameter [MD = −2.71, 95% CI: (−4.40, −1.03), *P* < 0.00001, *I*^2^ = 91%, *P* for heterogeneity=0.002]. Sequential exclusion of each study revealed that after removing the study by Li et al. (which had the largest weight), the recalculated effect was [MD = −2.03, 95% CI: (−3.84, −0.22), *P* < 0.00001, *I*^2^ = 92%, *P* = 0.03]; after excluding Wang et al., the result was [MD = −2.38, 95% CI: (−4.25, −0.51), *P* = 0.0001, *I*^2^ = 83%, *P* = 0.01]; and after simultaneously excluding both Li and Wang, heterogeneity decreased markedly [MD = −1.36, 95% CI: (−2.34, −0.37), *P* = 0.22, *I*^2^ = 32%, *P* = 0.007]. Compared with the original estimate, the direction of effect remained unchanged, and although the magnitude diminished, it retained statistical significance, suggesting that the finding of acupuncture-induced LVEDD reduction is generally robust. The initial high heterogeneity appeared to originate primarily from the studies by Li and Wang. Examination of the original study characteristics indicated that Li et al. administered intravenous dapoxetine prior to surgery alongside electroacupuncture and conventional care, whereas Wang et al. applied electroacupuncture pretreatment only 1–2 h before PCI. These differences in intervention timing and co-interventions—deviating from the post-PCI acupuncture plus routine care design in most other studies—likely constituted the main source of heterogeneity for this outcome.

**Figure 4:**
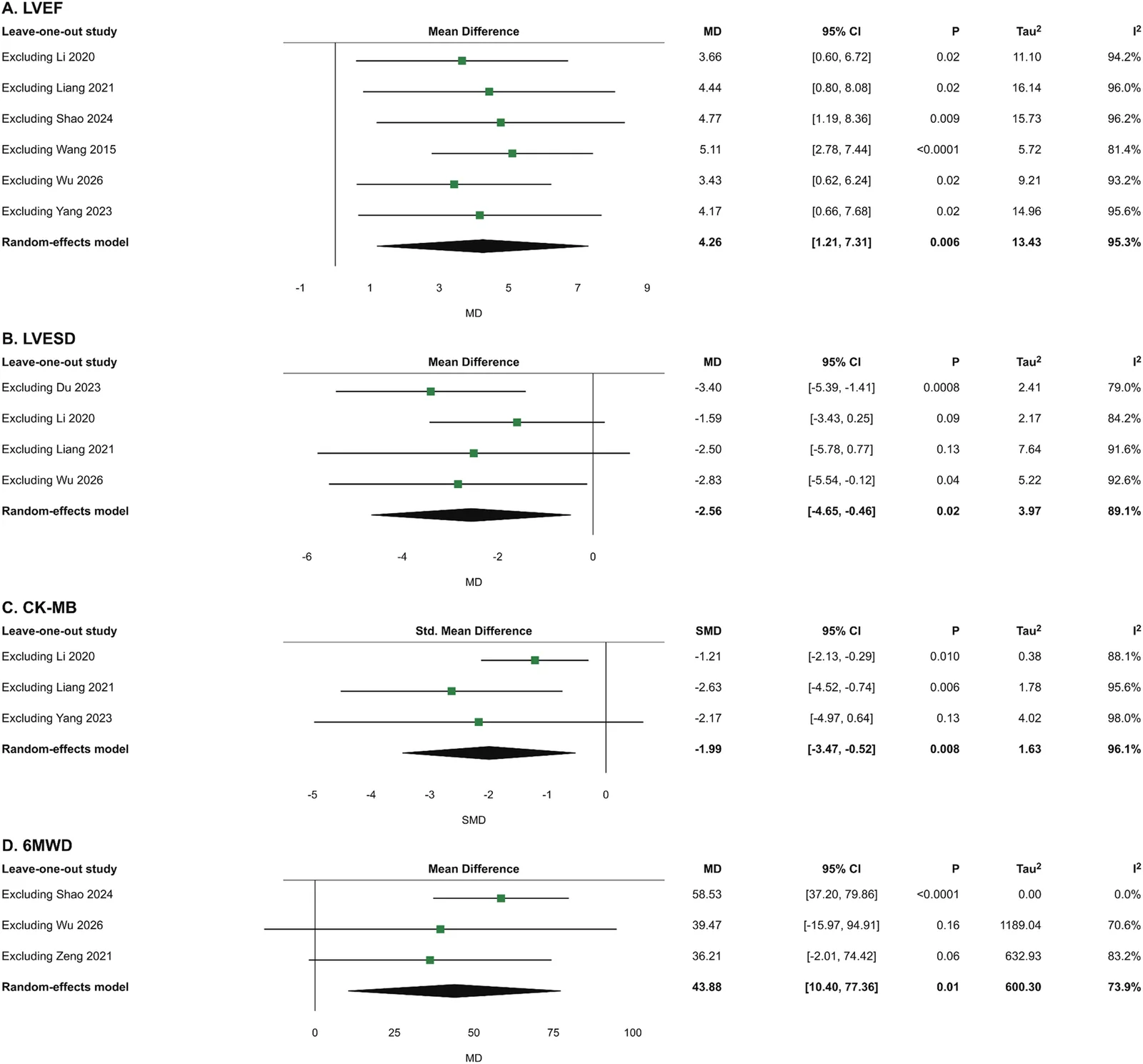
Leave-one-out sensitivity analyses of outcomes with substantial heterogeneity. **(A)** The effect of LVEF. **(B)** The effect of LVESD. **(C)** Serum CK-MB levels. **(D)** The effect on increasing 6MWD.

For other outcomes with high heterogeneity, including LVEF (*I*^2^ = 95%), LVESD (*I*^2^ = 89%), CK-MB (*I*^2^ = 96%), and 6MWD (*I*^2^ = 74%), similar leave-one-out sensitivity analyses were performed, with results presented in Figure 4.

Across all sensitivity analyses, the direction of the pooled effect did not reverse after excluding any single study, indicating satisfactory overall robustness. Nevertheless, it should be noted that for several indicators, *I*^2^ values remained partially above 50% even after removing primary sources of heterogeneity, suggesting residual heterogeneity possibly related to clinical variations across studies—such as differences in acupoint selection, treatment course duration, and timing of outcome measurements. These factors warrant standardization in future study designs.

### 3.5 Subgroup analysis and exploratory meta-regression

To further explore potential sources of heterogeneity, subgroup analyses were performed for outcomes reported in more than three studies and with relatively complete reporting of key clinical characteristics, including acupoint selection, acupuncture modality, timing of intervention, treatment duration, co-interventions, and timing of outcome assessment.

Given the substantial heterogeneity observed for LVEF, a subgroup analysis stratified by acupuncture modality was conducted. In the manual acupuncture subgroup (Liang, Shao, and Yang), heterogeneity was markedly reduced and the pooled effect remained statistically significant (MD = 3.44, 95% CI: 2.12–4.76; *I*^2^ = 39%, *P* for heterogeneity = 0.19; *P* for overall effect <0.00001). In contrast, heterogeneity remained high in the electroacupuncture subgroup (Li, Wang, and Wu), and the pooled effect was not statistically significant (MD = 5.29, 95% CI −0.56 to 11.14; *I*^2^ = 98%, *P* for heterogeneity <0.00001; *P* for overall effect = 0.08). These findings suggest that acupuncture modality may partly contribute to the heterogeneity in LVEF. However, the marked residual heterogeneity within the electroacupuncture subgroup indicates that other factors, such as treatment timing, duration, co-interventions, and peri-procedural management, may also have influenced the results. Therefore, these subgroup findings should be interpreted as exploratory.

In addition, exploratory meta-regression analyses were performed for the two primary outcomes that each included at least six studies, namely LVEF and LVEDD, to preliminarily assess whether study-level characteristics were associated with between-study heterogeneity in effect estimates (As shown in the Supplementary Table S4.). Because only six studies were available for each outcome (*k* = 6), multivariable meta-regression was not undertaken, as models including multiple covariates would be unstable and prone to overfitting. Univariate exploratory meta-regression showed that acupuncture modality, treatment duration, sample size, and mean age were not significantly associated with variation in the effect estimates for either LVEF or LVEDD; all corresponding *P* values were greater than 0.05, and all 95% confidence intervals crossed zero. These results suggest that the currently available study-level covariates are insufficient to adequately explain the observed heterogeneity. Given the limited number of included studies, these findings should be interpreted with caution.

### 3.6 GRADE evidence quality classification

The GRADE assessment showed that, due to methodological limitations, the certainty of evidence was moderate for MACE, low for left ventricular ejection fraction (LVEF), left ventricular end-diastolic diameter (LVEDD), and 6-minute walk distance (6MWD), and very low for serum CK-MB levels and left ventricular end-systolic diameter (LVESD) because of substantial heterogeneity and wide confidence intervals for the effect estimates. Detailed results are shown in Table 3. The detailed criteria for the GRADE assessment can be found in Supplementary Table S1.

**Table 3:** GRADE evidence profiles and summary of findings table.

Outcome	No. of RCTs	Risk of bias	Inconsistency	Indirectness	Imprecision	Other considerations	Sample size		Effect estimate (95% CI)	Certainty of evidence
							T	C		
LVEF	6	Downgraded one level^a^	Downgraded one level^b^	Not downgraded	Not downgraded	Not downgraded	302	302	MD = 4.26，95%CI (1.21，7.31)	⊕⊕◯◯ LOW
LVEDD	6	Downgraded one level^a^	Downgraded one level^b^	Not downgraded	Not downgraded	Not downgraded	320	321	MD = −1.36，95%CI (−2.34，−0.37)	⊕⊕◯◯ LOW
LVESD	4	Downgraded one level^a^	Downgraded one level^b^	Not downgraded	Downgraded one level c	Not downgraded	168	169	MD = −2.56，95%CI (−4.65，−0.46)	⊕◯◯◯ VERY LOW
CK-MB	3	Downgraded one level^a^	Downgraded one level^b^	Not downgraded	Downgraded one level c	Not downgraded	141	141	SMD=−1.99，95%CI (−3.46，−0.52)	⊕◯◯◯ VERY LOW
6MWD	3	Downgraded one level^a^	Downgraded one level^b^	Not downgraded	Not downgraded	Not downgraded	97	97	MD = 43.88，95%CI (10.40，77.36)	⊕⊕◯◯ LOW
MACE	3	Downgraded one level^a^	Not downgraded	Not downgraded	Downgraded one level c	Not downgraded	188	188	RR = 0.40，95%CI (0.24，0.66)	⊕⊕⊕◯ MODERATE

T, treatment group; C, control group.

a Allocation concealment and blinding were unclear (risk of bias).

b Substantial heterogeneity (inconsistency) (Downgraded by one level for inconsistency because, although statistical heterogeneity was substantial (*I*^2^ > 75%), the direction of effect was consistent across all studies and the heterogeneity arose predominantly from clinical diversity (e.g., different treatment durations and acupoint combinations) rather than contradictory effect estimates. Therefore, we judged the inconsistency downgraded one level.).c: Wide confidence interval for the effect estimate (imprecision).

## 4 Discussion

### 4.1 Summary of the main findings

In traditional medicine, coronary artery disease falls under the category of “chest obstruction” (Xiong Bi) and “heart obstruction” (Xin Bi). Acupuncture has been used to treat this condition for centuries with well-documented efficacy. Contemporary research recognizes acupuncture as a simple, low-risk intervention with unique advantages, playing an active role in the prevention and management of coronary heart disease (25). To the best of our knowledge, this is the first systematic comparison evaluating the efficacy of acupuncture for improving cardiac function in patients after PCI. The pooled findings of this meta-analysis suggest that, compared with conventional treatment alone, acupuncture combined with conventional treatment improves cardiac function indices, enhances exercise tolerance, reduces serum CK-MB levels, and decreases the incidence of MACE, with statistically differences for these outcomes. Specifically, the acupuncture group exhibited a significantly higher LVEF and greater reductions in LVESD, LVEDD, and serum CK-MB levels than the control group, suggesting that acupuncture may be associated with improved cardiac function and reduced markers of myocardial injury. Regarding exercise tolerance, the acupuncture group showed an increase in 6MWD, highlighting the promising potential of acupuncture in improving cardiac function in post-PCI patients.

### 4.2 Clinical basis and therapeutic mechanism

The eight included studies involved a total of 19 acupoints (the Huatuo Jiaji points, located 0.5 cun lateral to the posterior midline at the lower borders of the spinous processes from T1 to L5, are extra-meridian points, with 34 points bilaterally; these were counted as a single point for statistical purposes). The six most frequently used acupoints were Neiguan (PC6) (87.5%, 7/8), Xuehai (SP10) (37.5%, 3/8), Danzhong (CV17) (25%, 2/8), Shenmen (HT7) (25%, 2/8), Zusanli (ST36) (25%, 2/8), and Gongsun (SP4) (25%, 2/8). A diagram of the high-frequency acupoints is presented in SF1 of the Supplementary Material.

Neiguan (PC6), a point belonging to the Hand-Jueyin Pericardium Meridian, serving as its Luo-Connecting point and as one of the Eight Confluent Points was the most frequently used acupoint, both alone and in combination. Clinically, needling PC6 has achieved favorable effects in the prevention and treatment of various cardiovascular diseases. Preconditioning with acupuncture at PC6 has been shown to help maintain cardiomyocyte calcium homeostasis, enhance oxygen free radical scavenging capacity, and alleviate cardiac inflammation and functional injury in a narrative review (26). Additionally, needling PC6 can modulate the MAPK signaling pathway, thereby attenuating cardiomyocyte injury and ischemia-reperfusion injury. Yin et al. (27) concluded that acupuncture at PC6 can also inhibit detrimental neural signaling and protect cardiomyocytes by downregulating the expression of *β*3-adrenergic receptors. Xuehai (SP10), located superior to the medial epicondyle of the femur in the midportion of the vastus medialis muscle, belongs to the Spleen Meridian of Foot-Taiyin. It exerts dual-directional regulatory effects, including replenishing and activating blood and resolving stasis. Zhao et al. (28) demonstrated that needling SP10 can improve tongue appearance, hemorheological parameters, and coagulation profiles in patients with blood stasis syndrome, as well as regulate the release of vasoactive factors and accelerate microcirculation. Danzhong (CV17), located on the chest and belonging to the Conception Vessel, is the Front-Mu point of the Pericardium Meridian and the influential point for Qi. Studies (29, 30) suggest that CV17 acts on the heart through neuromodulatory mechanisms, improving coronary blood flow and myocardial ischemia, thereby relieving stable angina. Zhang et al. (31) further reported that combining CV17 with PC6 produced synergistic effects, improving cardiac structure and function in rats with chronic heart failure. Shenmen (HT7), located at the wrist and belonging to the Heart Meridian of Hand-Shaoyin, is the Yuan-Source point of the Heart. Numerous clinical studies (32) have confirmed that needling HT7 can improve symptoms and signs in patients with coronary heart disease through effects on myocardial electrical activity, heart rate, cardiac function, and blood metabolism. Lin et al. (33) observed that HT7 combined with PC6 effectively increased cardiac output and enhanced cardiac function in patients undergoing coronary artery bypass graft surgery. Zusanli (ST36), located on the anterolateral aspect of the lower leg, is the He-Sea point of the Stomach Meridian of Foot-Yangming. Zhang et al. (34) found in an animal study that electroacupuncture at ST36 primarily protected the myocardium by activating the cholinergic anti-inflammatory pathway, reducing high levels of TNF-α, MPO, and NO, and lowering serum CK-MB. Moreover, the combination of ST36 and PC6 exemplifies the traditional medicine principle of “combined use of root and branch acupoints.” Zhang et al. (35) demonstrated that concurrent electroacupuncture at ST36 and PC6 activated the AMPK/Nrf2 signaling pathway, inhibited Drp1-mediated excessive mitochondrial fission, and reduced mitochondrial dysfunction caused by fragmentation and vacuolization, thereby attenuating myocardial injury in MIRI rats. Gongsun (SP4), located on the medial border of the foot anterior and inferior to the base of the first metatarsal, also belongs to the Spleen Meridian of Foot-Taiyin, serving as its Luo-Connecting point and one of the Eight Confluent Points. In clinical practice, needling SP4 alone is relatively uncommon for cardiac conditions; it is usually combined with PC6 to co-regulate cardiac function. Cai et al. (36) experimentally demonstrated that needling SP4 combined with PC6 has relatively specific therapeutic effects on cardio-thoracic diseases, significantly elevating the depressed ST segment on the ECG in coronary heart disease. In summary, the selection of acupoints centered on Neiguan (PC6) may exert myocardial protection and improve cardiac function through multiple pathways, which could be the key mechanism underlying the improvements in cardiac function indices observed in post-PCI patients.

### 4.3 Limitations

This research faces a few key drawbacks. First, variations exist across studies in acupoint selection, intervention protocols, treatment timing and duration, and outcome assessments. Although subgroup analyses and random-effects models were applied to address these sources of heterogeneity, marked heterogeneity persisted across several endpoints. Specifically, heterogeneity in LVEF was likely attributable to the type of intervention (manual acupuncture vs. electroacupuncture), whereas that in LVESD was primarily associated with inconsistent timing of post-PCI intervention. The high heterogeneity in CK-MB probably stemmed from discrepancies in measurement units, and the heterogeneity in 6MWD was mainly linked to divergent assessment time points. Collectively, these factors limit the robustness of the pooled estimates.

Second, only eight RCTs were included, with a relatively modest total sample size, and merely one was published in English. The paucity of high-quality, large-scale, multinational trials, coupled with limited sample sizes, restricts the generalizability of acupuncture's effectiveness to post-PCI patients in non-Chinese regions and may introduce selection bias. Among the included studies, only one explicitly reported using a centralized randomization system, and the remainder did not mention allocation concealment or blinding methods. Given that only single-blinding is feasible in acupuncture trials, this raises the risk of selection, performance, and detection biases. Moreover, most studies failed to report sample size calculations, which may have undermined statistical power and the stability of the findings.

A further methodological constraint pertains to the use of two distinct control conditions: usual care alone, and usual care combined with sham acupuncture or sham electrostimulation. These two control strategies are fundamentally distinct both clinically and scientifically. A sham control is designed to control for non-specific factors—such as placebo effects, patient expectations, and the therapeutic context—thereby enabling a more precise estimation of acupuncture-specific effects. In contrast, comparison with usual care alone does not adequately disentangle the specific effects of needling at acupoints from broader contextual and non-specific influences. Given the very limited evidence from sham-controlled trials, it remains uncertain whether the observed benefits reflect a specific therapeutic effect of acupuncture or are, at least partially, attributable to non-specific placebo or contextual effects. This limitation should be carefully considered when interpreting the potential efficacy of acupuncture for post-PCI patients. Additionally, it is noteworthy that only one of the eight RCTs incorporated standardized cardiac rehabilitation—a class I intervention per international guidelines—into its protocol; the remaining seven trials administered acupuncture alongside conventional pharmacotherapy following PCI. Accordingly, the results of this meta-analysis are primarily applicable to acupuncture as an adjunct to standard medical therapy and do not support conclusions regarding acupuncture as a replacement for, or a direct comparator to, standardized cardiac rehabilitation.

Fourth, although subgroup analyses indicated improvements in LVEF and LVEDD, the mean age of participants ranged from 54.6 to 64.16 years in control groups and 56.55 to 64.16 years in intervention groups, predominantly encompassing middle-aged and older adults. No age-stratified results were available using the clinical thresholds of 60 or 65 years, nor was there systematic information on key geriatric variables such as frailty, multimorbidity, and polypharmacy. Consequently, the current evidence is only indirectly applicable to older patients undergoing PCI, and conclusions regarding acupuncture tolerability, clinical applicability in the context of multimorbidity, and rehabilitation benefits should be interpreted with caution.

Finally, another limitation of this systematic review is the inadequate reporting of safety outcomes. Although three included studies reported the incidence of major adverse cardiovascular events (MACE), none of the eight trials systematically documented or monitored acupuncture-related adverse events, such as needling syncope, local hematoma, bleeding, pain, or rare cases of pneumothorax. Therefore, the absence of reported acupuncture-related events in these studies should not be misinterpreted as an absence of risk. Given the lack of standardized safety assessments, definitive conclusions regarding the overall safety of acupuncture in post-PCI patients cannot be drawn at this time.

In summary, these limitations reduce the credibility of the pooled estimates, and the precise clinical value of acupuncture for patients following PCI warrants further investigation through high-quality studies.

## 5 Conclusion

In conclusion, acupuncture has possible benefit in improving cardiac function in post-PCI patients. Compared with conventional treatment alone, acupuncture plus conventional treatment increases LVEF and 6MWD, reduces LVEDD, LVESD, and CK-MB levels, and decreases the incidence of MACE. However, due to the small sample size, the general lack of allocation concealment and rigorous blinding, and the significant heterogeneity in several outcome measures, the current evidence is of limited quality. Future studies should adhere to the STRICTA (Standards for Reporting Interventions in Clinical Trials of Acupuncture) guidelines and employ rigorous designs with large sample sizes and standardized intervention protocols to further verify the therapeutic effect of acupuncture on cardiac function in patients after PCI and to provide more reliable evidence for its broader application.

## Data Availability

The original contributions presented in the study are included in the article/Supplementary Material, further inquiries can be directed to the corresponding author.
