# Supplementary material for: Can acupuncture improve cardiac function in patients after percutaneous coronary intervention? A systematic review and meta-analysis
Source: Front Med (Lausanne). 2026 Sep 9;13:1926216. doi: 10.3389/fmed.2026.1926216 (PMC13597401; doi:10.3389/fmed.2026.1926216)
Supplement: Supplementary file 1 [file Datasheet1.docx]

***Supplementary Material***

**1. Supplementary Tables**

**Supplementary Table S1. Full name of the abbreviation**

| Abbreviations | Full name |
| --- | --- |
| 6MWD | 6-minute walk distance |
| ACU | Acupuncture |
| BID | Bis in die |
| CK-MB  LVEF | serum creatine kinase-MB  left ventricular ejection fraction |
| CT | Conventional Treatment |
| EA | Electroacupuncture |
| LVESD | left ventricular end-systolic diameter Manual acupuncture |
| LVEDD | left ventricular end-diastolic diameter |
| MA | Manual acupuncture |
| MACE  PCI | major adverse cardiovascular events  percutaneous coronary intervention |
| QD | Quaque die |
| QOD | Quaque altera die |
| RCTs | Randomized Controlled Trials |
| SMD | Stand mean difference |
|  |  |

**Supplementary Table S2.** Preferred Reporting Items for Systematic Reviews and Meta-Analyses 2020 (PRISMA 2020) Checklists

PRISMA 2020 Main Checklist

| **Topic** | **No.** | **Item** | **Location where item is reported** |
| --- | --- | --- | --- |
| **TITLE** |  | Can Acupuncture Improve Cardiac Function in Patients After Percutaneous Coronary Intervention (PCI)? A Systematic Review and Meta‑Analysis |  |
| **Title** | 1 | A Systematic Review and Meta-Analysis |  |
| **ABSTRACT** |  |  |  |
| **Abstract** | 2 | See the PRISMA 2020 for Abstracts checklist |  |
| **INTRODUCTION** |  |  |  |
| **Ra****tionale** | 3 | Describe the rationale for the review in the context of existing knowledge. |  |
| **Objectives** | 4 | Provide an explicit statement of the objective(s) or question(s) the review addresses. |  |
| **METHODS** |  |  |  |
| **Eligibility criteria** | 5 | Specify the inclusion and exclusion criteria for the review and how studies were grouped for the syntheses. | Methods 2.2 |
| **Information sources** | 6 | Specify all databases, registers, websites, organisations, reference lists and other sources searched or consulted to identify studies. Specify the date when each source was last searched or consulted. | Methods 2.1 |
| **Search strategy** | 7 | Present the full search strategies for all databases, registers and websites, including any filters and limits used. | Methods 2.1 |
| **Selection process** | 8 | Specify the methods used to decide whether a study met the inclusion criteria of the review, including how many reviewers screened each record and each report retrieved, whether they worked independently, and if applicable, details of automation tools used in the process. | Methods 2.3 |
| **Data collection process** | 9 | Specify the methods used to collect data from reports, including how many reviewers collected data from each report, whether they worked independently, any processes for obtaining or confirming data from study investigators, and if applicable, details of automation tools used in the process. | Methods 2.3 |
| **Data items** | 10a | List and define all outcomes for which data were sought. Specify whether all results that were compatible with each outcome domain in each study were sought (e.g. for all measures, time points, analyses), and if not, the methods used to decide which results to collect. | Methods 2.2 & 2.3 |
|  | 10b | List and define all other variables for which data were sought (e.g. participant and intervention characteristics, funding sources). Describe any assumptions made about any missing or unclear information. | Methods 2.3 |
| **Study risk of bias assessment** | 11 | Specify the methods used to assess risk of bias in the included studies, including details of the tool(s) used, how many reviewers assessed each study and whether they worked independently, and if applicable, details of automation tools used in the process. | Methods 2.4 |
| **Effect measures** | 12 | Specify for each outcome the effect measure(s) (e.g. risk ratio, mean difference) used in the synthesis or presentation of results. | Methods 2.5 |
| **Synthesis methods** | 13a | Describe the processes used to decide which studies were eligible for each synthesis (e.g. tabulating the study intervention characteristics and comparing against the planned groups for each synthesis (item 5)). | Methods 2.2 & 2.3; Results 3.1 |
|  | 13b | Describe any methods required to prepare the data for presentation or synthesis, such as handling of missing summary statistics, or data conversions. | Methods 2.5 |
|  | 13c | Describe any methods used to tabulate or visually display results of individual studies and syntheses. | Methods 2.5; Tables 1-2; Figure 3 |
|  | 13d | Describe any methods used to synthesize results and provide a rationale for the choice(s). If meta-analysis was performed, describe the model(s), method(s) to identify the presence and extent of statistical heterogeneity, and software package(s) used. | Methods 2.5 |
|  | 13e | Describe any methods used to explore possible causes of heterogeneity among study results (e.g. subgroup analysis, meta-regression). | Methods 2.5; Results 3.5 |
|  | 13f | Describe any sensitivity analyses conducted to assess robustness of the synthesized results. | Methods 2.5; Results 3.4 |
| **Reporting bias assessment** | 14 | Describe any methods used to assess risk of bias due to missing results in a synthesis (arising from reporting biases). | Methods 2.5 |
| **Certainty assessment** | 15 | Describe any methods used to assess certainty (or confidence) in the body of evidence for an outcome. | Methods 2.4; Results 3.6 |
| **RESULTS** |  |  |  |
| **Study selection** | 16a | Describe the results of the search and selection process, from the number of records identified in the search to the number of studies included in the review, ideally using a flow diagram. | Results 3.1; Figure 1 |
|  | 16b | Cite studies that might appear to meet the inclusion criteria, but which were excluded, and explain why they were excluded. | Results 3.1; Supplemental Table S3 |
| **Study characteristics** | 17 | Cite each included study and present its characteristics. | Results 3.1; Tables 1&2 |
| **Risk of bias in studies** | 18 | Present assessments of risk of bias for each included study. | Results 3.2; Figure 2 |
| **Results of individual studies** | 19 | For all outcomes, present, for each study: (a) summary statistics for each group (where appropriate) and (b) an effect estimate and its precision (e.g. confidence/credible interval), ideally using structured tables or plots. | Results 3.3; Figure 3 |
| **Results of syntheses** | 20a | For each synthesis, briefly summarise the characteristics and risk of bias among contributing studies. | Results 3.2 & 3.3 |
|  | 20b | Present results of all statistical syntheses conducted. If meta-analysis was done, present for each the summary estimate and its precision (e.g. confidence/credible interval) and measures of statistical heterogeneity. If comparing groups, describe the direction of the effect. | Results 3.3; Figure3 |
|  | 20c | Present results of all investigations of possible causes of heterogeneity among study results. | Results 3.4 & 3.5 |
|  | 20d | Present results of all sensitivity analyses conducted to assess the robustness of the synthesized results. | Results 3.4 |
| **Reporting biases** | 21 | Present assessments of risk of bias due to missing results (arising from reporting biases) for each synthesis assessed. | Methods 2.5 |
| **Certainty of evidence** | 22 | Present assessments of certainty (or confidence) in the body of evidence for each outcome assessed. | Results 3.6; Table 3 |
| **DISCUSSION** |  |  |  |
| **Discussion** | 23a | Provide a general interpretation of the results in the context of other evidence. | Discussion 4.1 |
|  | 23b | Discuss any limitations of the evidence included in the review. | Discussion 4.3 |
|  | 23c | Discuss any limitations of the review processes used. | Discussion 4.3 |
|  | 23d | Discuss implications of the results for practice, policy, and future research. | Discussion4.3; Conclusion |
| **OTHER INFORMATION** |  |  |  |
| **Registration and protocol** | 24a | Provide registration information for the review, including register name and registration number, or state that the review was not registered. | Abstract & Methods |
|  | 24b | Indicate where the review protocol can be accessed, or state that a protocol was not prepared. | Methods (PROSPERO registration) |
|  | 24c | Describe and explain any amendments to information provided at registration or in the protocol. | Not applicable |
| **Support** | 25 | Describe sources of financial or non-financial support for the review, and the role of the funders or sponsors in the review. | Not applicable |
| **Competing interests** | 26 | Declare any competing interests of review authors. |  |
| **Availability of data, code and other materials** | 27 | Report which of the following are publicly available and where they can be found: template data collection forms; data extracted from included studies; data used for all analyses; analytic code; any other materials used in the review. | Availability of data and materials |

**PRIMSA Abstract Checklist**

| **Topic** | **No.** | **Item** | **Reported?** |
| --- | --- | --- | --- |
| **TITLE** |  |  |  |
| **Title** | 1 | Identify the report as a systematic review. | Yes |
| **BACKGROUND** |  |  |  |
| **Objectives** | 2 | Provide an explicit statement of the main objective(s) or question(s) the review addresses. | Yes |
| **METHODS** |  |  |  |
| **Eligibility criteria** | 3 | Specify the inclusion and exclusion criteria for the review. | Yes |
| **Information sources** | 4 | Specify the information sources (e.g. databases, registers) used to identify studies and the date when each was last searched. | Yes |
| **Risk of bias** | 5 | Specify the methods used to assess risk of bias in the included studies. | Yes |
| **Synthesis of results** | 6 | Specify the methods used to present and synthesize results. | Yes |
| **RESULTS** |  |  |  |
| **Included studies** | 7 | Give the total number of included studies and participants and summarise relevant characteristics of studies. | Yes |
| **Synthesis of results** | 8 | Present results for main outcomes, preferably indicating the number of included studies and participants for each. If meta-analysis was done, report the summary estimate and confidence/credible interval. If comparing groups, indicate the direction of the effect (i.e. which group is favoured). | Yes |
| **DISCUSSION** |  |  |  |
| **Limitations of evidence** | 9 | Provide a brief summary of the limitations of the evidence included in the review (e.g. study risk of bias, inconsistency and imprecision). | Yes |
| **Interpretation** | 10 | Provide a general interpretation of the results and important implications. | Yes |
| **OTHER** |  |  |  |
| **Funding** | 11 | Specify the primary source of funding for the review. | Yes |
| **Registration** | 12 | Provide the register name and registration number. | Yes |

*From:* Page MJ, McKenzie JE, Bossuyt PM, Boutron I, Hoffmann TC, Mulrow CD, et al. The PRISMA 2020 statement: an updated guideline for reporting systematic reviews. MetaArXiv. 2020, September 14. DOI: 10.31222/osf.io/v7gm2. For more information, visit: www.prisma-statement.org

**Supplemental Table S3**. Search strategy

| **Database** | PubMed |
| --- | --- |
| **Date Searched** | 30 May 2026 |
| **Search Terms** | All Fields (“acupuncture” OR “electroacupuncture” OR “acupoint” OR “warm needling” OR “acupoint injection” OR “fire acupuncture” OR “wrist-ankle acupuncture” OR “auricular acupuncture”) AND (“Percutaneous Coronary Intervention” OR “Percutaneous Coronary Revascularization” OR “Coronary Interventions, Percutaneous” OR “Interventions, Percutaneous Coronary” OR “PCI” OR “after coronary stenting” OR “after coronary artery intervention surgery” OR “coronary heart disease”) |
| **Results Returned** | 44 |

| **Database** | Ovid |
| --- | --- |
| **Date Searched** | 30 May 2026 |
| **Search Terms** | ("Percutaneous Coronary Intervention" or "Percutaneous Coronary Revascularization" or "Coronary Interventions, Percutaneous" or "Interventions, Percutaneous Coronary" or "PCI" or "after coronary stenting" or "after coronary artery intervention surgery" or "coronary heart disease").mp. and (acupunct or electroacupunct or electro-acupunct or acupoint or "warm needle" or "acupoint injection" or "fire acupunct" or "fire needle" or (wrist adj2 ankle adj3 acupunct) or "auricular acupunct" or "ear acupunct").af. [mp=ti, ab, hw, tc, id, ot, tm, mf, tx, bt, ct, sh, nm, fx, kf, ox, px, rx, an, ui, ds, on, sy, ux, mx] |
| **Results Returned** | 10 |

| **Database** | EMBASE |
| --- | --- |
| **Date Searched** | 30 May 2026 |
| **Search Terms** | ('all fields' OR (all AND fields AND ('acupuncture'/exp OR 'acupuncture' OR 'electroacupuncture'/exp OR 'electroacupuncture' OR 'acupoint'/exp OR 'acupoint' OR 'warm needling'/exp OR 'warm needling' OR 'acupoint injection'/exp OR 'acupoint injection' OR 'fire acupuncture'/exp OR 'fire acupuncture' OR 'wrist-ankle acupuncture' OR 'auricular acupuncture'/exp OR 'auricular acupuncture'))) AND ('percutaneous coronary intervention'/exp OR 'percutaneous coronary intervention' OR 'percutaneous coronary revascularization'/exp OR 'percutaneous coronary revascularization' OR 'coronary interventions, percutaneous' OR 'interventions, percutaneous coronary' OR 'pci' OR 'after coronary stenting' OR 'after coronary artery intervention surgery' OR 'coronary heart disease'/exp OR 'coronary heart disease') |
| **Results Returned** | 53 |

| **Database** | Cochrane Library |
| --- | --- |
| **Date Searched** | 30 May 2026 |
| **Search Terms** | "Acupuncture OR electroacupuncture OR acusector OR acupoint OR auricular in Title Abstract Keyword AND Percutaneous Coronary Intervention OR Percutaneous Coronary Revascularization OR Coronary Interventions, Percutaneous OR Interventions, Percutaneous Coronary OR after coronary stenting OR after coronary artery intervention surgery in Title Abstract Keyword" |
| **Results Returned** | 296 |

| **Database** | China National Knowledge Infrastructure |
| --- | --- |
| **Date Searched** | 30 May 2026 |
| **Search Terms** | (针刺 + 针灸 + 电针 + 穴刺激 + 腕踝针 + 耳针 + 揿针) AND (经皮冠状动脉介入 + PCI + 冠状动脉支架植入+ 冠脉支架术后+冠脉介入术后+经皮冠状动脉血运重建) (Title/Abstract/Keyword) |
| **Results Returned** | 105 |

| **Database** | VIP Database |
| --- | --- |
| **Date Searched** | 30 May 2026 |
| **Search Terms** | (经皮冠状动脉介入术 OR PCI OR 冠状动脉介入术后 OR 冠脉支架术后) AND (针刺 OR 针灸 OR 电针 OR 火针 OR 温针灸 OR 耳针 OR 腕踝针 OR 穴位注射 OR 腧穴)（题名或关键词） |
| **Results Returned** | 29 |

| **Database** | Sinomed Database |
| --- | --- |
| **Date Searched** | 30 May 2026 |
| **Search Terms** | "针刺"[全部字段] OR "针灸"[全部字段] OR "电针"[全部字段] OR "火针"[全部字段] OR "温针灸"[全部字段] OR "耳针"[全部字段]OR "腕踝针"[全部字段] AND OR "穴位注射"[全部字段] OR "腧穴"[全部字段] AND AND "经皮冠状动脉介入术"[全部字段]OR "PCI"[全部字段] OR"冠状动脉介入术后"[全部字段]OR"冠脉支架术后"[全部字段] |
| **Results Returned** | 2 |

| **Database** | WANFANG Medical |
| --- | --- |
| **Date Searched** | 30 May 2026 |
| **Search Terms** | （经皮冠状动脉介入术 OR PCI OR 冠状动脉介入术后 OR 冠脉支架术后) AND(针刺 OR 针灸 OR 电针 OR 火针 OR 温针灸 OR 耳针 OR 腕踝针 OR 穴位注射 OR 腧穴） |
| **Results Returned** | 218 |

**Supplementary Table 3.** Excluded studies and the reason for exclusion.

| Serial number | Study  （in Chinese） | Study  （in English） | Reason for exclusion |
| --- | --- | --- | --- |
| 1 | 吴双,夏铭徽,张宏如 & 姜文.基于脉络学说指导的电针干预对STEMI-PCI术后短期心室重构影响的随机对照临床研究.针刺研究,1-9.https://doi.org/10.13702/j.1000-0607.20251241. | Wu Shuang, Xia Minghui, Zhang Hongru & Jiang Wen. A Randomized Controlled Clinical Study on the Effect of Electroacupuncture Intervention Guided by Meridian Theory on Short-Term Ventricular Remodeling after STEMI-PCI. Acupuncture Research, 1-9. https://doi.org/10.13702/j.1000-0607.20251241. | No relevant outcome data reported |
| 2 | 肖燕,丁亮 & 马丹.(2025).电针辅助治疗对经皮冠状动脉介入术后心肌缺血再灌注损伤患者炎症因子、MG53、sCD40L和Lp-PLA2水平的影响.上海针灸杂志,44(12),1462-1467.https://doi.org/10.13460/j.issn.1005-0957.2025.14.0006. | Xiao Yan, Ding Liang & Ma Dan. (2025). The influence of electroacupuncture-assisted treatment on the levels of inflammatory factors, MG53, sCD40L and Lp-PLA2 in patients with myocardial ischemia-reperfusion injury after percutaneous coronary intervention. Shanghai Journal of Acupuncture and Moxibustion, 44(12), 1462-1467. https://doi.org/10.13460/j.issn.1005-0957.2025.14.0006. | control group was ineligible |
| 3 | 张迪,李鸿鹏,马江,聂谦,孙剑峰,吴志鹏... & 赵珏.(2023).眼针带针运动对经皮冠状动脉介入术治疗患者术后心率变异性及预后的影响研究.中国全科医学,26(36),4535-4544. | Zhang Di, Li Hongpeng, Ma Jiang, Nie Qian, Sun Jianfeng, Wu Zhipeng... & Zhao Jue. (2023). Study on the Impact of Eye Needling Needle Movement on Postoperative Heart Rate Variability and Prognosis in Patients Treated with Percutaneous Coronary Intervention. Chinese General Practice, 26(36), 4535-4544. | No relevant outcome data reported |
| 4 | 褚敏,高彦彩,李磊 & 吕海燕.(2025).活血散风针刺法对冠心病伴高血压患者PCI围术期血压、术后血管内皮功能及冠脉再狭窄风险的影响.辽宁中医杂志,52(5),97-100.https://doi.org/10.13192/j.issn.1000-1719.2025.05.027. | Chu Min, Gao Yancai, Li Lei & Lu Haiyan. (2025). The effect of the acupoint needling therapy of promoting blood circulation and dispelling wind on blood pressure, postoperative vascular endothelial function and the risk of coronary re-stenosis in patients with coronary heart disease and hypertension during PCI. Liaoning Journal of Traditional Chinese Medicine, 52(5), 97-100. https://doi.org/10.13192/j.issn.1000-1719.2025.05.027. | No relevant outcome data reported |
| 5 | 史海阳,郭新斌 & 李圣耀.(2023).太极拳结合针灸对急性心肌梗死PCI术后病人心率变异性、心功能及生活质量的影响.护理研究,37(7),1309-1313. | Shi Haiyang, Guo Xinbin & Li Shengyao. (2023). The Impact of Tai Chi Combined with Acupuncture on Heart Rate Variability, Cardiac Function and Quality of Life in Patients after PCI for Acute Myocardial Infarction. Nursing Research, 37(7), 1309-1313. | Intervention was ineligible |
| 6 | 凌发样.针刺辅助治疗AMI-PCI术后患者的临床疗效评价及外泌体miRNA差异表达研究[D].成都中医药大学,2024.DOI:10.26988/d.cnki.gcdzu.2024.000037. | Clinical efficacy of traditional Chinese medicine combined with acupuncture on chronic heart failure (cardiac function grade II) with Qi deficiency and blood stasis syndrome | control group was ineligible |
| 7 | 赵方超,高岩林,蔡华山,等.经皮穴位电刺激对经皮冠状动脉介入术后患者血管内皮功能及炎性因子的影响[J].针刺研究,2021,46(8):684-689.DOI:10.13702/j.1000-0607.200784. | Zhao Fangchao, Gao Yanlin, Cai Huashan, et al. Effects of Percutaneous Acupoint Electrical Stimulation on Vascular Endothelial Function and Inflammatory Factors in Patients after Percutaneous Coronary Intervention [J]. Acupuncture Research, 2021, 46(8): 684-689. DOI: 10.13702/j.1000-0607.200784. | No relevant outcome data reported |
| 8 | 毛立伟,季鹏,余萍,等.运动训练结合揿针治疗对中老年冠心病患者PCI术后运动能力、心肺功能及生存质量的影响[J].中国康复医学杂志,2019,34(8):920-925. | Mao Liwei, Ji Peng, Yu Ping, et al. The influence of exercise training combined with acupuncture treatment on the exercise capacity, cardiopulmonary function and quality of life of middle-aged and elderly patients with coronary heart disease after PCI [J]. Chinese Journal of Rehabilitation Medicine, 2019, 34(8): 920-925. | Intervention was ineligible |
| 9 | 周仕鸿.康复训练结合针灸疗法对痰瘀互结型冠心病患者PCI术后的临床疗效观察[D].成都中医药大学,2017. | Zhou Shihong. Clinical Efficacy Observation of Rehabilitation Training Combined with Acupuncture Therapy on Patients with Tumour-Stasis Interlocking Type Coronary Heart Disease after PCI Surgery [D]. Chengdu University of Traditional Chinese Medicine, 2017. | Intervention was ineligible |
| 10 | 王婷婷.针刺结合运动疗法对冠心病PCI术后患者生存质量的影响[D].湖北中医药大学,2016. | Wang Tingting. The Impact of Acupuncture Combined with Exercise Therapy on the Quality of Life of Patients with Coronary Heart Disease after PCI Surgery [D]. Hubei University of Chinese Medicine, 2016. | Intervention was ineligible |
| 11 | 谢亚宁.电针预处理对经皮冠状动脉支架术患者心肌保护作用的研究[D].第四军医大学,2015. | Xie Yaning. Research on the Protective Effect of Electroacupuncture Pre-treatment on Myocardium in Patients Undergoing Percutaneous Coronary Stent Surgery [D]. Fourth Military Medical University, 2015. | control group was ineligible |
| 12 | 张子扬.TEAS在急性心肌梗死PCI术后心肌缺血再灌注损伤中的疗效与机制研究[D].长春中医药大学,2025.DOI:10.26980/d.cnki.gcczc.2025.000082. | Zhang Ziyang. The Efficacy and Mechanism of TEAS in Acute Myocardial Infarction PCI-Induced Myocardial Ischemia and Reperfusion Injury [D]. Changchun University of Traditional Chinese Medicine, 2025. DOI: 10.26980/d.cnki.gcczc.2025.000082. | control group was ineligible |
| 13 | 李鸿鹏.基于ERAS理论的眼针带针运动疗法对中老年冠心病PCI术后患者心脏康复疗效的临床随机对照研究[D].成都中医药大学,2024.DOI:10.26988/d.cnki.gcdzu.2024.000639. | Li Hongpeng. A Clinical Randomized Controlled Study on the Effect of Eye Needle-Attached Needle Movement Therapy Based on ERAS Theory on Cardiac Rehabilitation of Middle-aged and Elderly Patients after PCI for Coronary Heart Disease [D]. Chengdu University of Traditional Chinese Medicine, 2024. DOI: 10.26988/d.cnki.gcdzu.2024.000639. Effect of Enhanced External Counterpulsation Combined with Acupuncture on Cardiac Function in Elderly Patients with Chronic HF | Intervention was ineligible |
| 14 | 张涨.黄帝内针对冠心病PCI术后气虚血瘀型患者炎症因子及临床预后的影响[D].广西中医药大学,2020.DOI:10.27879/d.cnki.ggxzy.2020.000283. | Zhang Zhang. The Impact of Huangdi Neijing on Inflammatory Factors and Clinical Prognosis in Patients with Qi Deficiency and Blood Stasis Syndrome after PCI for Coronary Heart Disease [D]. Guangxi University of Chinese Medicine, 2020. DOI: 10.27879/d.cnki.ggxzy.2020.000283. | No relevant outcome data reported |
| 15 | 魏X、彭Y、王K、克里格T、严S、吴F、范M、周J。电针治疗经皮冠状动脉介入术患者急性心肌梗死的慢血流/无血流：一项前瞻性随机对照试验。Front Cardiovasc Med. 2026年3月10日；13:1756414。doi: 10.3389/fcvm.2026.1756414。PMID: 41890868；PMCID: PMC13014618。 | Wei X, Peng Y, Wang K, Krieg T, Yan S, Wu F, Fan M, Zhou J. Electroacupuncture for slow flow/no-reflow in patients with acute myocardial infarction undergoing percutaneous coronary intervention: a pilot randomized controlled trial. Front Cardiovasc Med. 2026 Mar 10;13:1756414. doi: 10.3389/fcvm.2026.1756414. PMID: 41890868; PMCID: PMC13014618. | No relevant outcome data reported |
| 16 | 张Z、李M、王Y、张X、古R、 Rao C、李T、李M、李X、王J、陈N、孙H、李Z、王H。经皮电神经刺激（TENS）用于STEMI患者PCI术后缺血再灌注损伤及二级预防：一项随机对照试验的研究方案。Trials. 2026年3月23日；27(1):338. doi: 10.1186/s13063-026-09632-4. PMID: 41872950; PMCID: PMC13130640. | Zhang Z, Li M, Wang Y, Zhang X, Gou R, Rao C, Li T, Li M, Li X, Wang J, Chen N, Sun H, Li Z, Wang H. Transcutaneous electrical nerve stimulation (TENS) for ischemia-reperfusion injury and secondary prevention post-PCI in STEMI: study protocol for a randomized controlled trial. Trials. 2026 Mar 23;27(1):338. doi: 10.1186/s13063-026-09632-4. PMID: 41872950; PMCID: PMC13130640. | No original data |
| 17 | 彭Y、魏X、吴F、范M、王K、周J。针灸治疗经皮冠状动脉介入术患者急性心肌梗死中缓慢血流/无再灌注现象：一项前瞻性随机对照试验方案。Front Cardiovasc Med. 2024年6月18日；11:1401269。doi: 10.3389/fcvm.2024.1401269。PMID: 38957330；PMCID: PMC11218819。 | Peng Y, Wei X, Wu F, Fan M, Wang K, Zhou J. Electroacupuncture for slow flow/no-reflow phenomenon in patients with acute myocardial infarction undergoing percutaneous coronary intervention: protocol for a pilot randomized controlled trial. Front Cardiovasc Med. 2024 Jun 18;11:1401269. doi: 10.3389/fcvm.2024.1401269. PMID: 38957330; PMCID: PMC11218819. | No original data |
| 18 | 沈 H，陈 YJ。[电针联合全身麻醉对冠心病高血糖患者围手术期心肌损伤的影响]。中华中西医结合杂志。2012年12月；32(12):1607-10。PMID: 23469596。 | Shen H, Chen YJ. [Effects of electro-acupuncture combined general anesthesia on myocardial injury of high blood sugar patients with coronary heart disease in the perioperative phase]. Zhongguo Zhong Xi Yi Jie He Za Zhi. 2012 Dec;32(12):1607-10. Chinese. PMID: 23469596. | Intervention was ineligible |
| 19 | 赵Y、林F、秦Y、谢W、齐W、倪Q、张H、韩M、赵J、刘L、胡L、肖J、李W、梁F、郑Q。针灸作为经皮冠状动脉介入治疗（PCI）后ST段抬高型心肌梗死（STEMI）患者辅助治疗的效果与安全性：一项随机对照试验的研究方案。BMC补充医学疗法。2024年8月14日；24(1):306。doi: 10.1186/s12906-024-04608-w。PMID: 39143484；PMCID: PMC11325820。 | Zhao Y, Ling F, Qin Y, Xie W, Qi W, Nie Q, Zhang H, Han M, Zhao J, Liu L, Hu L, Xiao J, Li W, Liang F, Zheng Q. The effect and safety of acupuncture as adjunctive therapy for STEMI patients after PCI: study protocol of a randomized controlled trial. BMC Complement Med Ther. 2024 Aug 14;24(1):306. doi: 10.1186/s12906-024-04608-w. PMID: 39143484; PMCID: PMC11325820. | No original data |
| 20 | 张N、谢B、杜J、高Q。针灸与运动康复对经皮冠状动脉介入术后急性冠脉综合征患者生活质量的影响。《美国转化研究杂志》。2024年6月15日；16(6):2662-2669。doi: 10.62347/KIFR7353。PMID: 39006291；PMCID: PMC11236627。 | Zhang N, Xie B, Du J, Gao Q. Effect of acupuncture and exercise rehabilitation on quality of life in acute coronary syndrome patients after percutaneous coronary intervention. Am J Transl Res. 2024 Jun 15;16(6):2662-2669. doi: 10.62347/KIFR7353. PMID: 39006291; PMCID: PMC11236627. | Intervention was ineligible |
| 21 | 倪X、谢Y、王Q、钟H、陈M、王F、熊L。经皮电针刺激对小儿心脏患者的心脏保护作用：一项随机对照临床试验。《小儿麻醉学》2012年8月；22(8):805-11。doi: 10.1111/j.1460-9592.2012.03822.x。2012年3月2日在线发表。PMID: 22380768。 | Ni X, Xie Y, Wang Q, Zhong H, Chen M, Wang F, Xiong L. Cardioprotective effect of transcutaneous electric acupoint stimulation in the pediatric cardiac patients: a randomized controlled clinical trial. Paediatr Anaesth. 2012 Aug;22(8):805-11. doi: 10.1111/j.1460-9592.2012.03822.x. Epub 2012 Mar 2. PMID: 22380768. | No relevant outcome data reported |
| 22 | 杨L、王Q、陈M、卢Z、陈S、熊L。电针预处理对接受心脏瓣膜置换手术患者的心脏保护作用：一项随机对照试验。Ann Thorac Surg. 2010年3月；89(3):781-6. doi: 10.1016/j.athoracsur.2009.12.003. PMID: 20172127. | Yang L, Yang J, Wang Q, Chen M, Lu Z, Chen S, Xiong L. Cardioprotective effects of electroacupuncture pretreatment on patients undergoing heart valve replacement surgery: a randomized controlled trial. Ann Thorac Surg. 2010 Mar;89(3):781-6. doi: 10.1016/j.athoracsur.2009.12.003. PMID: 20172127. | No relevant outcome data reported |

**Supplementary Table S4.** Exploratory univariate meta-regression analysis

|  | Covariate | Univariate analysis | | | |  | |
| --- | --- | --- | --- | --- | --- | --- | --- |
|  |  | β | 95% CI | *P* | τ² |  | |
| LVEF | Method | 1.928 | -3.466～7.322 | 0.484 | 10.265 | |  |
|  | Duration | 0.208 | -0.627～1.043 | 0.625 | 10.425 | |  |
|  | Sample size | -0.244 | -0.728～0.239 | 0.321 | 8.631 | |  |
|  | Mean age | -0.231 | -1.128～0.667 | 0.614 | 10.453 | |  |
| LVEDD | Method | -2.632 | -5.544～0.279 | 0.076 | 2.621 | |  |
|  | Duration | 0.516 | -0.076～1.108 | 0.087 | 2.797 | |  |
|  | Sample size | -0.189 | -0.574～0.197 | 0.337 | 4.375 | |  |
|  | Mean age | 0.381 | -0.292～-1.055 | 0.267 | 4.051 | |  |

**Abbreviations:** LVEF, left ventricular ejection fraction; LVEDD, left ventricular end-diastolic diameter; β, regression coefficient; CI, confidence interval; τ², heterogeneity variance.

**2. Supplementary** **Figure**

**Supplementary Figure F1.** Schematic diagram of the most frequently selected acupoints.

**Annotation:**CV17:Danzhong(25%, 2/8),PC6 :Neiguan(87.5%, 7/8), HT7: Shenmen(25%, 2/8), SP10: Xuehai(37.5%, 3/8),ST36: Zusanli(25%, 2/8), SP4:Gongsun(25%, 2/8).

**Supplementary Appendix S1 GRADE evidence evaluation criteria**

For each outcome, the certainty of evidence was assessed using the GRADE framework across five domains that may decrease certainty: risk of bias, inconsistency, indirectness, imprecision, and publication bias. Randomized controlled trial evidence was initially rated as high certainty and observational evidence as low certainty; each serious limitation led to downgrading by 1 level and each very serious limitation led to downgrading by 2 levels, yielding a final rating of high, moderate, low, or very low certainty. Certainty could also be upgraded for a large magnitude of effect, a dose-response gradient, or when all plausible residual confounding would reduce the observed effect.

**Risk of bias** was judged by the overall methodological limitations of the included studies, including random sequence generation, allocation concealment, blinding of participants and personnel, blinding of outcome assessment, incomplete outcome data, selective reporting, and other potential biases using the Cochrane RoB 2.0 tool (for RCTs). Downgrading was applied based on the proportion of pooled weight contributed by studies with moderate-to-high risk of bias: **No serious risk** when low-risk studies accounted for ≥70% of the total pooled weight; **Serious risk (−1 level)** when moderate-to-high risk studies accounted for 30%–70% of the total pooled weight; **Very serious risk (−2 levels)** when high-risk studies accounted for >70% of the total pooled weight, or when the outcome was subjective and none of the included studies employed blinding.

**Inconsistency** was assessed by statistical heterogeneity (I² statistic and Q-test), consistency in the direction of effects, and overlap of confidence intervals. I² was used as a primary indicator but interpreted in conjunction with directionality: **No serious risk** when I² ≤ 40% and Q-test *P* > 0.10; **Serious risk (−1 level)** when 40% < I² ≤ 75%; however, if all effect estimates were directionally consistent (all point estimates lay on the same side of the null in the forest plot), the downgrade could be reduced by one level (e.g., from −1 to 0, or from −2 to −1); **Very serious risk (−2 levels)** when I² > 75%, unless all effect directions were consistent, in which case the downgrade could be reduced to −1; if effect directions were contradictory (some favored intervention, some favored control), the full −2 downgrade was maintained.

**Indirectness** was judged according to the PICO framework when there were important differences between the available evidence and the target population, intervention, comparator, or outcomes. Each of the four PICO elements was evaluated separately: **No serious risk** when all four elements aligned with the review question; **Serious risk (−1 level)** when two elements were misaligned; **Very serious risk (−2 levels)** when three or more elements were misaligned, or when a historical control was used instead of a concurrent comparator. The use of surrogate outcomes was considered a deviation in the outcome element.

**Imprecision** was mainly evaluated using the width of the 95% confidence interval, whether it crossed the no-effect threshold (null value of 1.0 for ratio measures), and whether the sample size reached the optimal information size (OIS). A dual-criteria approach was applied: **No serious risk** when total events (for dichotomous outcomes) ≥300 (or total sample size ≥400) AND the 95% CI did not cross the null value AND the ratio of the upper to lower CI limits was ≤3; **Serious risk (−1 level)** when total events <300 OR the 95% CI crossed the null with an upper-to-lower CI limit ratio >3; **Very serious risk (−2 levels)** when the 95% CI crossed the null with an upper-to-lower CI limit ratio >5.

**Publication bias** was assessed using funnel plot asymmetry, Egger's regression test when appropriate (≥10 studies), trial registration information, and the likelihood of missing negative studies, with statistical tests interpreted in the context of the entire evidence base. A mandatory safeguard for small meta-analyses was applied: **No serious risk** when the number of included studies was ≥10 and Egger's test *P* > 0.05, with no visible funnel plot asymmetry; **Serious risk (−1 level)** when the number of included studies was <10 (regardless of Egger's test result, due to low statistical power for detecting asymmetry), or when 0.01 < *P* ≤ 0.05 for studies ≥10; **Very serious risk (−2 levels)** when Egger's test *P* ≤ 0.01 for studies ≥10.

Publication bias was not formally assessed because each outcome included fewer than 10 studies, making funnel plot asymmetry and Egger’s test unreliable.

**Factors that may increase the level of quality of evidence** included a large magnitude of effect, a dose-response gradient, and the direction of plausible residual confounding.

**Magnitude of effect** was assessed using odds ratio (OR) or relative risk (RR): **No upgrade** when 0.5 < effect estimate < 2; **Upgrade +1 level** when 2 ≤ effect estimate < 5 or 0.2 < effect estimate ≤ 0.5, provided the 95% CI was not excessively wide (i.e., did not meet the "Very serious imprecision" threshold); **Upgrade +2 levels** when effect estimate ≥5 or ≤0.2, provided the 95% CI was not excessively wide. Upgrading was prohibited if the 95% CI was excessively wide or heterogeneity was substantial (I² > 75%).

**Plausible confounding** was considered for upgrade only when all known and unmeasured residual confounding would systematically underestimate the true treatment effect (i.e., the true effect is likely larger than reported). If the direction of residual confounding was ambiguous, no upgrade was applied. This factor could upgrade the certainty by a maximum of 1 level.

**Dose-response gradient** was evaluated when a statistically significant linear dose-response relationship was present across studies or within a study (*P* for trend < 0.05), which could provide an additional upgrade of 1 level.

The **final certainty** was derived by applying all downgrades and upgrades to the initial baseline. The four levels were: **High**, **Moderate**, **Low**, and **Very Low**. A ceiling rule was applied: RCTs could achieve a maximum of "High," while observational studies could achieve a maximum of "Moderate" (exceptions to upgrade observational studies to "High" were permitted only in the presence of an extremely large effect size with a clear dose-response gradient and negligible confounders, and required explicit justification). A floor rule was also applied: the lowest assignable level was "Very Low," with no further downgrading beyond this point.

If the number of downgrades and upgrades were numerically balanced, the authors could assign an intermediate grade (e.g., "Moderate to High"), with explicit justification provided in the text. For any domain rated as "Very serious risk"—particularly high heterogeneity (I² > 75%)—pre-specified sensitivity analyses (e.g., leave-one-out meta-analysis, subgroup analysis, or meta-regression) were performed to explore the source of variance, and the final downgrade decision was based on these sensitivity results.

All assessments were conducted independently by two reviewers, and disagreements were resolved through consensus or arbitration by a third reviewer. The specific reasoning for each downgrade or upgrade was explicitly reported in the summary of findings table.
